# Salvage Radiotherapy versus Observation for Biochemical Recurrence following Radical Prostatectomy for Prostate Cancer: A Matched Pair Analysis

**DOI:** 10.3390/cancers14030740

**Published:** 2022-01-31

**Authors:** Derya Tilki, Felix Preisser, Reinhard Thamm, Raisa S. Pompe, Felix K.-H. Chun, Markus Graefen, Alessandra Siegmann, Dirk Böhmer, Volker Budach, Thomas Wiegel

**Affiliations:** 1 Martini-Klinik Prostate Cancer Center, University Hospital Hamburg Eppendorf, 20251 Hamburg, Germany; rapompe@gmail.com (R.S.P.); graefen@uke.de (M.G.); 2 Department of Urology, University Hospital Hamburg-Eppendorf, 20251 Hamburg, Germany; 3 Department of Urology, Koc University Hospital, Istanbul 34450, Turkey; 4 Department of Urology, University Hospital Frankfurt, 60590 Frankfurt, Germany; Felix.Preisser@kgu.de (F.P.); Felix.Chun@kgu.de (F.K.-H.C.); 5 Department of Radiotherapy and Radiooncology, University Hospital Ulm, 89081 Ulm, Germany; Rheinhard.thamm@uniklinik-ulm.de (R.T.); Thomas.Wiegel@uniklinik-ulm.de (T.W.); 6 Department of Radiation Oncology, Charité University Hospital, 10117 Berlin, Germany; Alessandra.siegmann@charite.de (A.S.); Dirk.boehmer@charite.de (D.B.); Volker.budach@charite.de (V.B.)

**Keywords:** SRT, radical prostatectomy, salvage radiotherapy, oncological outcome, metastasis-free survival, death

## Abstract

**Simple Summary:**

Salvage radiotherapy improves oncologic outcomes in prostate cancer patients who develop biochemical recurrence after radical prostatectomy. However, the evidence on hard clinical endpoints is scarce. Within this study, we compare the long-term oncologic outcomes of patients with biochemical recurrence after prostatectomy, who were treated with either salvage radiotherapy or no radiotherapy. Our results show that patients who were treated with salvage radiotherapy after the development of biochemical recurrence following radical prostatectomy had a lower risk of developing metastasis and lower risk of death within the follow-up. These findings further underline the curative potential of salvage radiotherapy in the case of biochemical recurrence after radical prostatectomy, and should be discussed with these patients.

**Abstract:**

Background: Salvage radiotherapy (SRT) improves oncologic outcomes in prostate cancer (PCa) patients who develop biochemical recurrence (BCR) after radical prostatectomy (RP). However, evidence on hard clinical endpoints is scarce. We compare long-term oncologic outcomes of SRT versus no radiotherapy (noRT) in patients with BCR after RP. Patients and methods: Within a multi-institutional database, we identified patients with BCR after RP between 1989 and 2016 for PCa. Patients with lymph node invasion, with adjuvant radiotherapy, or with additional androgen deprivation therapy at BCR were excluded. In all patients with SRT, SRT was delivered to the prostatic bed only. Propensity score matching (PSM) was performed to account for differences in pathologic tumor characteristics. Kaplan–Meier analyses and Cox regression models tested the effect of SRT versus no RT on metastasis-free (MFS) and overall survival (OS). Results: Of 1832 patients with BCR, 32.9% *(n* = 603) received SRT without ADT. The median follow-up was 95.9 months. Median total SRT dose was 70.2 Gy. After 1:1 PSM, at 15 years after RP, MFS and OS rates were 84.3 versus 76.9% (*p* < 0.001) and 85.3 versus 74.4% (*p* = 0.04) for SRT and noRT, respectively. In multivariable Cox regression models, SRT was an independent predictor for metastasis (HR: 0.37, *p* < 0.001) and OS (HR: 0.64, *p* = 0.03). Conclusion: This is the first matched-pair analysis investigating the impact of SRT versus observation only in post-RP recurrent PCa. After compensating for established risk factors, SRT was associated with better long-term MFS and OS. These results on clinical endpoints underline the curative potential of SRT.

## 1. Introduction

After radical prostatectomy (RP), prostatic specific antigen (PSA) is the cornerstone for follow-up of prostate cancer (PCA) patients [1,2]. In the case of rising or persisting PSA levels with the development of biochemical recurrence (BCR) after RP, salvage radiotherapy (SRT) is associated with a better long-term oncologic outcome compared with observation [3,4]. These observations are mainly based on two studies: within a cohort of 635 men, Trock et al. reported a reduction in prostate cancer specific mortality for SRT versus observation [5]. Similarly, Cotter and colleagues reported a reduction in all-cause mortality through SRT, within a cohort of 519 patients who developed BCR after RP [6].

Owing to the lack of available studies regarding a long-term outcome, we investigated the relationship between SRT versus observation after RP with the development of BCR and clinical endpoints, within a large database derived from three high-volume institutions. Specifically, we investigated the impact between the use of SRT versus observation after BCR and metastasis-free survival (MFS) and overall survival (OS).

## 2. Patients and Methods

### 2.1. Study Population

After Institutional Review Board approval, patients that underwent RP (1989–2016), who developed BCR, defined as two consecutive PSA rises above the post-RP nadir, were identified from three high-volume centers (Charité University Hospital, Berlin, Germany, University Hospital Ulm, Ulm, Germany and Martini-Klinik Prostate Cancer Center, Germany).

Patients with post-RP persisting or recurring PSA were stratified according to post-BCR observation without androgen deprivation therapy (ADT) (no SRT performed) versus SRT without ADT before or simultaneously with SRT. The decision to undergo SRT was at the discretion of the patient and treating urologist. Information on PSA-doubling time was unavailable for the study period. Patients with lymph node invasion (pN+) and patients with adjuvant radiotherapy were excluded. These selection criteria yielded 1832 patients, which represented the focus of the current analysis.

### 2.2. Radiotherapy

SRT with a median dose of 70.2 Gy (interquartile range [IQR]: 66.2–72.0 Gy, 1.8/2.0 Gy per fraction) was delivered to the prostatic bed and for pT3b-pT4 additionally to the former seminal vesicles without any additional ADT. The pelvic lymphatics were not irradiated. Radiotherapy modalities comprised 3D-conformal radiotherapy (N = 369) and, since 2006, fixed intensity modulated radiotherapy (IMRT, N = 52) and rotational IMRT (N = 182) including image-guided RT in contemporary patients.

### 2.3. Outcomes

Metastasis was diagnosed by positive imaging after BCR. Imaging procedures after PSA increase consisted of bone scan and/or computed tomography and/or abdominal magnetic resonance imaging and/or 11C-choline positron emission tomography/computed tomography scan (PET/CT) and/or 18F-choline PET/CT and/or Ga-68-PSMA PET/CT. MFS was calculated as time from RP to metastasis or last follow-up. OS was calculated as time from RP to death or last follow-up.

### 2.4. Statistical Analyses

Descriptive statistics included frequencies and proportions for categorical variables. Medians and interquartile ranges were reported for continuously coded variables. The Chi-square test examined the statistical significance in proportions’ differences. The Mann–Whitney U test examined the significance of medians’ differences.

A 1:1 propensity score matching (PSM) was performed to test the impact of SRT versus observation after BCR on MFS and OS [7,8,9]. Matching was performed for tumor and patients’ characteristics, namely, age, year of surgery, pathologic Gleason grade group (ISUP grade groups), surgical margin status, pathologic tumor stage, and preoperative PSA-value. After PSM, with the use of a caliper of 0.05, no significant differences between tumor characteristics were recorded between patients who were observed after BCR versus patients with SRT (Table 1), defined by a standardized mean difference (SMD) of <0.2.

Kaplan–Meier analyses depicted MFS and OS rates. Subsequently, two sets of multivariable Cox regression models were fitted to test the relationship between the performance of SRT and the oncologic outcomes, namely, MFS and OS. Specifically, the first set tested the relationship between SRT and MFS and the second tested the relationship between SRT and OS. All multivariable Cox models were performed after PSM and were adjusted for year of surgery, age, pathologic Gleason grade group, pathologic tumor stage, preoperative PSA, and positive margin status. A sensitivity analysis was performed at six months.

R software environment for statistical computing and graphics (version: 3.4.4) was used for all statistical analyses. All tests were two-sided with a level of significance set at *p* < 0.05.

## 3. Results

### 3.1. Descriptive Statistics

Of 1832 patients with BCR, 32.9% (*n* = 603) received SRT. The median follow-up of the entire cohort was 95.9 months. Patients managed with SRT more frequently harbored positive margins (41.6 vs. 24.8%, *p* < 0.001), had higher median pre-RP PSA (9.5 vs. 8.4 ng/mL, *p* < 0.001), and more frequently had organ-confined disease (53.7 vs. 45.3%, *p* < 0.001) and Gleason grade group 4 (12.9 vs. 1.9%, *p* < 0.001). Median PSA at the start of SRT was 0.3 ng/mL, while the median and mean time from RP to SRT was 25 months (IQR: 10–47 months) and 33 months, respectively.

### 3.2. Impact of SRT on Metastasis

**Figure 1:**
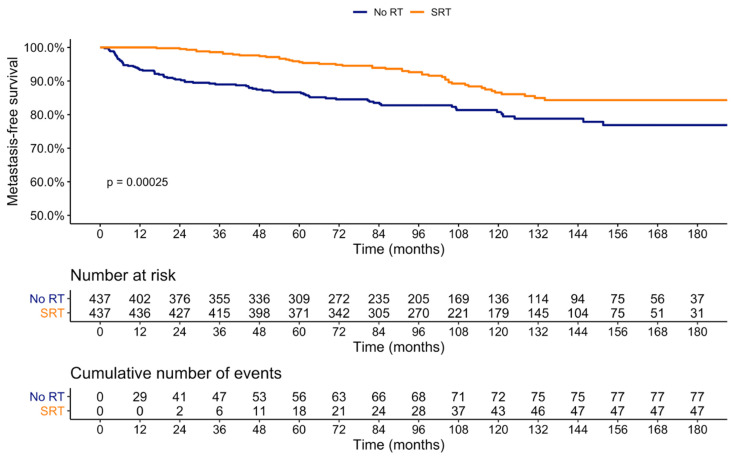
Kaplan–Meier plot depicting metastasis-free survival rates in prostate cancer patients treated with radical prostatectomy stratified according to observation vs. SRT for BCR, after 1:1 propensity score matching.

Overall, 105 patients developed metastases. After 1:1 PSM, at 15 years after RP, MFS rates were 84.3 versus 76.9% (*p* < 0.001) for SRT and noRT, respectively (Figure 1). In multivariable Cox regression models testing the relationship between SRT and development of metastasis (Table 2), SRT was an independent predictor (hazard ratio [HR]: 0.37, 95% confidence interval [95%-CI] 0.25–0.53, *p* < 0.001). Additionally, more contemporary year of surgery (HR: 1.22, 95%-CI: 1.16–1.28, *p* < 0.001), older age (HR: 0.97, 95%-CI: 0.94–0.99, *p* = 0.01), pathologic stage T3a (HR: 2.24, 95%-CI: 1.42–3.52, *p* < 0.001), pathologic stage T3b (HR: 4.01, 95%-CI: 2.41–6.67, *p* < 0.001), pathologic stage T4 (HR: 5.98, 95%-CI: 1.93–18.6, *p* < 0.01), Gleason grade group 3 (HR: 2.02, 95%-CI: 1.05–3.92, *p* = 0.03), and Gleason grade group 4–5 (HR: 4.16, 95%-CI: 2.13–8.11, *p* < 0.001) were all independent predictors for the development of metastasis.

Similar results were recorded in sensitivity analysis at six months. Here, at 15 years after RP, MFS rates were 84.3 versus 80.0% (*p* = 0.02) for SRT and no RT, respectively (Figure S1). In multivariable Cox regression models testing the relationship between SRT and the development of metastasis (Table S1), SRT was still an independent predictor (HR: 0.46, 95%-CI: 0.31–0.68, *p* < 0.001).

### 3.3. Impact of SRT on Survival

**Figure 2:**
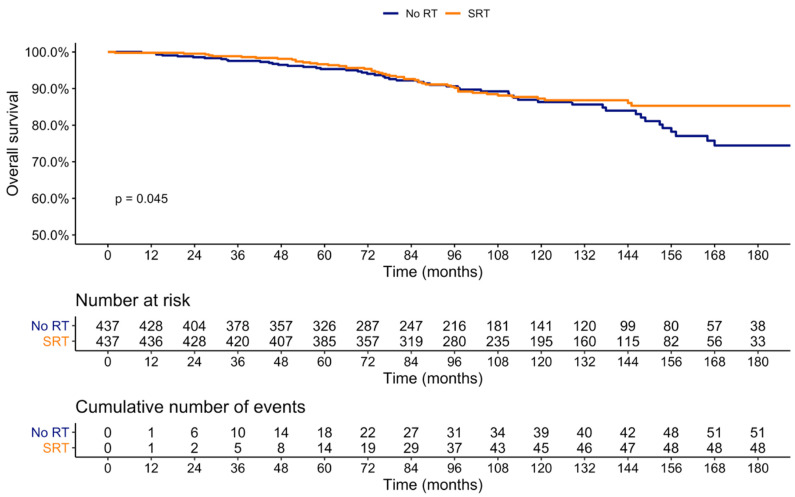
Kaplan–Meier plot depicting overall survival rates in prostate cancer patients treated with radical prostatectomy stratified according to observation vs. SRT for BCR, after 1:1 propensity score matching.

Overall, 108 men died during the reported period. After 1:1 PSM, at 15 years after RP, OS rates were 85.3 versus 74.4% (*p* = 0.04) for SRT and no RT, respectively (Figure 2). In multivariable Cox regression models predicting death (Table 3), SRT was an independent predictor (HR: 0.64, 95%-CI 0.43–0.96, *p* = 0.03). Additionally, pathologic stage T3a (HR: 1.93, 95%-CI: 1.14–3.26, *p* = 0.01), pathologic stage T3b (HR: 3.68, 95%-CI: 2.11–6.42, *p* < 0.001), pathologic stage T4 (HR: 3.18, 95%-CI: 1.17–8.65, *p* = 0.02), Gleason grade group 3 (HR: 2.16, 95%-CI: 1.15–4.06, *p* = 0.02), Gleason grade group 4–5 (HR: 2.82, 95%-CI: 1.48–5.37, *p* < 0.01), and older age (HR: 1.04, 95%-CI: 1.01–1.07, *p* = 0.04) were all independent predictors for death.

Similar results were recorded in sensitivity analysis at six months. Here, at 15 years after RP, OS rates were 85.5 versus 74.4% (*p* = 0.03) for SRT and noRT, respectively (Supplementary Figure S2). In multivariable Cox regression models testing the relationship between SRT and death (Supplementary Table S1), SRT was still an independent predictor (HR: 0.62, 95%-CI: 0.42–0.93, *p* = 0.02).

## 4. Discussion

SRT provides the possibility of a cure for patients with an increasing PSA after RP. Ref. [1] Several retrospective series demonstrated that SRT leads to a reduction in PSA during further follow-up in more than 50% of patients [10,11,12,13]. However, evidence of a survival benefit due to SRT in comparison with observation without SRT in patients with BCR after RP is lacking [5,6].

To improve the state of evidence based on solid endpoints, we investigated in a propensity-matched approach the long-term MFS and OS of patients with post-RP biochemically recurrent disease, who had either post-RP observation alone or received SRT.

At 15 years after RP, MFS rates of propensity matched patients were 84.3% versus 76.9% (*p* < 0.001) for SRT versus observation. It is noteworthy that, despite the development of BCR post-RP, even patients without SRT, but with favorable prognostic factors can have such a good long-term MFS. However, MFS may be prone to underdetection and underreporting.

An advantage from SRT versus observation was also observed in Kaplan–Meier curves after propensity score matching for OS with 85.3% versus 74.4% (*p* = 0.04) at 15 years. Moreover, in multivariable Cox regression models, SRT was an independent predictor for lower risk of developing metastases and death after RP. In line with these findings, all results were confirmed in sensitivity analyses performed at six months.

The favorable effect of SRT on OS is in line with data by Cotter et al., who additionally report on the dependence of the therapy outcome on the post-RP PSA doubling time (PSADT) and on comorbidity [2]. However, in that retrospective study, treatment procedures included SRT with concurrent or sequential ADT (16% in total), and only 25% of the patients received SRT alone [6].

This said, our results confirm the potential survival benefit of SRT within a more homogenous cohort, where no ADT was given in addition to SRT.

The late divergence of the OS curves (at ≥10 years of follow-up) in our cohort is not unusual and underlines the efficacy of RP alone and its considerable period of disease control, which has been previously reported [14]. Even in RP patients who develop BCR, Pompe et al. reported an overall mortality of only 20% at a median follow-up of 121 months [15]. This also reflects a good selection of surgical candidates.

Trock et al. reported much higher rates of metastasis than we observed in men with untreated BCR [5]. However, many of these patients had unfavorable characteristics such as locally advanced disease (60%), Gleason grade group 4–5 (33%), and even positive lymph nodes (30%). Moreover, considerable differences in the risk characteristics of patients in the three treatment arms observation (N = 397) versus SRT (N = 160) versus SRT + ADT (N = 78) were not compensated for. Active treatments limited the incidence of metastatic progression to 20% (SRT + ADT) and 27% (SRT), while in the observation arm, it was 45%, after a median and maximum post-RP follow-up of 9 years and 23 years, respectively. Their reporting period ended in 2007, suggesting that there were tangible differences in the treatment standards compared with more contemporary procedures [1].

A study on the natural history of patients with BCR showed a reduction in MFS of around 5% per year among 450 patients who never received any type of salvage treatment [16]. However, another study reported that the risk in untreated post-RP BCR patients to progress to a metastatic state correlates with the absolute PSA-level at time of BCR [17].

Additionally, the Gleason grade group had a significant impact on MFS in SRT-patients [18]. Beside clinical parameters that may be summarized in CAPRA-S, genomic classifiers such as the Decipher score have been suggested as predictors of MFS [19]. Recently, the expression of specific proteins during immunohistochemical analysis such as serine/arginine splicing factor 1 (SRSF-1) or microvascular density (MVD) has also been reported to be associated with a worse prognosis in prostate cancer patients [20]. While we could well confirm the impact of clinical risk factors for both MFS and OS, molecular markers or protein expression profiles were not investigated in our patients.

Our risk-adjusted study extends the evidence for the favorable impact of SRT for post-RP BCR. However, owing to the retrospective setting, no standardized follow-up sequences are available, which hampers, e.g., the reliable determination of parameters such as PSADT. The retrospective and tri-institutional setting may cause heterogeneity, e.g., in treatment techniques or the reporting of events, specifically of metastases. Despite the use of a matched-pair analysis, the retrospective design does not exclude unknown confounders, which may have influenced decision-making for or against the use of SRT. Ideally, a prospective randomized trial should be used to account for confounders. While there are three randomized trials comparing adjuvant radiotherapy to SRT—the RADICALS trial (Radiation Therapy and Androgen Deprivation Therapy in Treating Patients Who Have Undergone Surgery for Prostate Cancer trial—NCT00541047), the RAVES trial (Radiotherapy—Adjuvant Versus Early Salvage—NCT00860652), and the GETUG-AFU 16 trial (NCT00423475)—to the best of our knowledge, no prospective trial testing the oncologic benefit of SRT versus observation is currently in recruitment or being planned [21]. As it is highly unlikely that such a trial will be conducted in the near future, retrospective studies provide the best available evidence.

The long period of the study span may have influenced the results by itself. During the study period, several changes in radiotherapy modalities and/or surgical techniques may have occurred. Regarding MFS, changes in imaging modalities might have influenced our results. It is reasonable to assume that, in contemporary patients, more advanced imaging modalities with a higher sensitivity to detect metastasis have been used. However, detailed information on performed imaging for each patient was unavailable. Additionally, the lack of individual comorbidities is another limitation of our study. Thus, we were not able to account for it in our analysis. Indeed, comorbidities may be crucial in decision-making. However, as all patients underwent RP with a curative intent before development of BCR, it can be assumed that most included patients in our study had been potential candidates for SRT.

It was beyond the scope of the current analysis to weigh the advantages from SRT against the risk of overtreatment or of toxicity. Moreover, the potential of additional ADT for patients with a high-risk profile could not be considered.

## 5. Conclusions

This is the first matched-pair analysis investigating the impact of SRT versus observation in post-RP recurrent PCa. After compensating for established risk factors in our large cohort of patients, SRT was associated with better long-term MFS and OS in patients with BCR after RP. These results on clinical endpoints improve the level of evidence for the curative potential of SRT.

## Figures and Tables

**Table 1:** Descriptive tumor characteristics of RP patients with BCR stratified according to no RT and SRT, before and after 1:1 matching.

	Before 1:1 Matching			After 1:1 Matching		
	No RT (*n* = 1229)	SRT (*n* = 603)	SMD	No SRT (*n* = 437)	SRT (*n* = 437)	SMD
Age, mean (sd)	64.51 (6.3)	63.43 (6.2)	0.172	63.6 (6.5)	63.8 (6.1)	0.032
Year of surgery, mean (sd)	2005 (6.4)	2004 (4.8)	0.125	2005 (5.2)	2004 (4.7)	0.077
PSA, ng/mL, mean (sd)	11.76 (11.43)	12.96 (12.19)	0.102	12.1 (11.9)	12.1 (10.8)	0.001
Pathologic Gleason, n (%)			0.774			0.028
GG1	167 (13.6)	207 (34.3)		130 (29.7)	131 (30.0)	
GG2	615 (50.1)	183 (30.3)		165 (37.8)	165 (37.8)	
GG3	353 (28.7)	94 (15.6)		90 (20.6)	87 (19.9)	
GG4	23 (1.9)	78 (12.9)		17 (3.9)	19 (4.3)	
GG5	70 (5.7)	41 (6.8)		35 (8.0)	35 (8.0)	
Pathologic stage, n (%)			0.205			0.056
pT2	557 (45.3)	324 (53.7)		229 (52.4)	227 (51.9)	
pT3a	383 (31.2)	181 (30.0)		132 (30.2)	136 (31.1)	
pT3b	266 (21.6)	89 (14.8)		67 (15.3)	68 (15.6)	
pT4	23 (1.9)	9 (1.5)		9 (2.1)	6 (1.4)	
Positive surgical margin, n (%)	305 (24.8)	251 (41.6)	0.443	155 (35.5)	167 (38.2)	0.057

Abbreviations: GG—Gleason grade group; PSA—prostatic specific antigen; RP—radical prostatectomy; SD—standardized difference; SMD—standardized mean difference; SRT—salvage radiotherapy.

**Table 2:** Uni- and multivariable Cox regression predicting metastasis of patients with biochemical recurrence after radical prostatectomy and 1:1 matching, stratified according to salvage radiotherapy vs. observation.

	Univariable		Multivariable	
	HR (95%-CI)	*p*-Value	HR (95%-CI)	*p*-Value
No sRT (reference)	-	-	-	-
sRT	0.52 (0.36–0.74)	<0.001	0.37 (0.25–0.53)	<0.001
Year of surgery	1.21 (1.16–1.27)	<0.001	1.22 (1.16–1.28)	<0.001
Age	0.99 (0.97–1.02)	0.7	0.97 (0.94–0.99)	0.01
Preoperative PSA	1.01 (0.99–1.02)	0.2	0.99 (0.98–1.02)	0.9
Pathologic stage ≤ T2c (reference)	-	-	-	-
Pathologic stage T3a	2.36 (1.54–3.62)	<0.001	2.24 (1.42–3.52)	<0.001
Pathologic stage T3b	4.02 (2.55–6.34)	<0.001	4.01 (2.41–6.67)	<0.001
Pathologic stage T4	3.33 (1.19–9.34)	0.02	5.98 (1.93–18.6)	<0.01
GG1 (reference)	-	-	-	-
GG2	2.34 (1.31–4.18)	<0.01	1.50 (0.82–2.76)	0.2
GG3	4.91 (2.72–8.89)	<0.001	2.02 (1.05–3.92)	0.03
GG4–5	8.58 (4.72–15.6)	<0.001	4.16 (2.13–8.11)	<0.001
Positive surgical margin	0.77 (0.53–1.13)	0.2	0.72 (0.49–1.07)	0.1

Abbreviations: GG—Gleason grade group; sRT—salvage radiotherapy; PSA—prostatic specific antigen value.

**Table 3:** Uni- and multivariable Cox regression predicting death of patients with biochemical recurrence after radical prostatectomy and 1:1 matching, stratified according to salvage radiotherapy vs. observation.

	Univariable		Multivariable	
	HR (95%-CI)	*p*-Value	HR (95%-CI)	*p*-Value
No sRT (reference)	-	-	-	-
sRT	0.68 (0.46–0.99)	0.04	0.64 (0.43–0.96)	0.03
Year of surgery	0.98 (0.94–1.03)	0.4	0.99 (0.95–1.05)	0.9
Age	1.04 (1.01–1.07)	0.04	1.04 (1.01–1.07)	0.04
Preoperative PSA	1.02 (1.01–1.03)	<0.001	1.01 (0.99–1.02)	0.2
Pathologic stage ≤ T2c (reference)	-	-	-	-
Pathologic stage T3a	2.57 (1.57–4.22)	<0.001	1.93 (1.14–3.26)	0.01
Pathologic stage T3b	5.51 (3.31–9.15)	<0.001	3.68 (2.11–6.42)	<0.001
Pathologic stage T4	4.96 (1.90–12.9)	<0.01	3.18 (1.17–8.65)	0.02
GG1 (reference)	-	-	-	-
GG2	1.58 (0.92–2.69)	0.1	1.42 (0.81–2.47)	0.2
GG3	3.07 (1.73–5.46)	<0.001	2.16 (1.15–4.06)	0.02
GG4–5	4.63 (2.55–8.41)	<0.001	2.82 (1.48–5.37)	<0.01
Positive surgical margin	1.75 (1.19–2.56)	<0.01	1.38 (0.93–2.06)	0.1

Abbreviations: GG—Gleason grade group; sRT—salvage radiotherapy; PSA—prostatic specific antigen value.

## Data Availability

The datasets generated during and/or analyzed during the current study are available from the corresponding author on reasonable request.

## Supplementary Materials

The following supporting information can be downloaded at https://www.mdpi.com/article/10.3390/cancers14030740/s1, Figure S1: Kaplan–Meier plot depicting overall survival rates in prostate cancer patients treated with radical prostatectomy stratified according to observation vs. SRT for BCR, after 1:1 propensity score matching, sensitivity analysis at six months; Table S1: Multivariable Cox regression predicting metastasis and death of patients with biochemical recurrence after radical prostatectomy and 1:1 matching, stratified according to salvage radiotherapy vs. observation, sensitivity analysis at 6 months.
