# Supplementary material for: Salvage Radiotherapy versus Observation for Biochemical Recurrence following Radical Prostatectomy for Prostate Cancer: A Matched Pair Analysis
Source: Cancers (Basel). 2022 Jan 31;14(3):740. doi: 10.3390/cancers14030740 (PMC8833698; doi:10.3390/cancers14030740)
Supplement: Supplementary file 1 [file cancers-14-00740-s001.zip › cancers-1555131-supplementary.pdf]

Supplementary material

Table S1. Multivariable Cox regression predicting metastasis and death of patients with biochemical recurrence after radical prostatectomy and 1:1 matching, stratified according to salvage radiotherapy vs. observation, sensitivity analysis at 6 months.

	**Metastasis**		**Death**	
	**HR (95%-CI)**	**p-value**	**HR (95%-CI)**	**p-value**
No sRT (reference)	-	-	-	-
sRT	0.46 (0.31-0.68)	<0.001	0.62 (0.42-0.93)	0.02
Year of surgery	1.19 (1.13-1.25)	<0.001	0.99 (0.95-1.05)	0.9
Age	0.97 (0.94-0.99)	0.02	1.03 (0.99-1.07)	0.1
Preoperative PSA	1.01 (0.98-1.02)	0.9	1.01 (0.99-1.02)	0.2
Pathologic stage ≤T2c (reference)	-	-	-	-
Pathologic stage T3a	2.13 (1.32-3.44)	<0.01	2.02 (1.19-3.43)	<0.01
Pathologic stage T3b	3.67 (2.13-6.32)	<0.001	3.89 (2.22-6.84)	<0.001
Pathologic stage T4	5.33 (1.72-16.6)	<0.01	3.29 (1.21-8.99)	0.02
GG1 (reference)	-	-	-	-
GG2	1.56 (0.85-2.86)	0.2	1.40 (0.80-2.45)	0.2
GG3	1.89 (0.95-3.73)	0.1	2.04 (1.08-3.85)	0.03
GG4-5	3.59 (1.79-7.22)	<0.001	2.79 (1.46-5.31)	<0.01
Positive surgical margin	0.85 (0.56-1.28)	0.4	1.41 (0.95-2.11)	0.1

Abbreviations: GG – Gleason grade group; sRT – salvage radiotherapy; PSA – prostatic specific antigen value;.

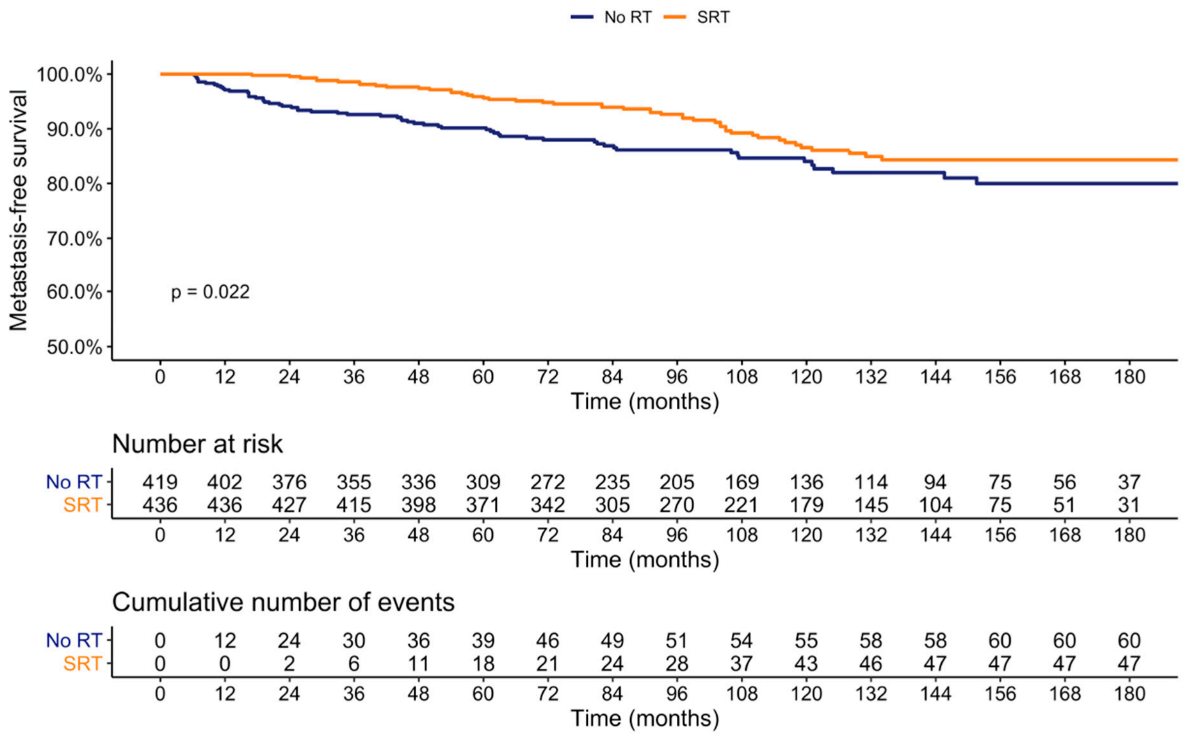

Figure S1. Kaplan-Meier plot depicting metastasis free survival rates in prostate cancer patients treated with radical prostatectomy stratified according to observation vs. SRT for BCR, after 1:1propensity score matching, sensitivity analysis at six months.

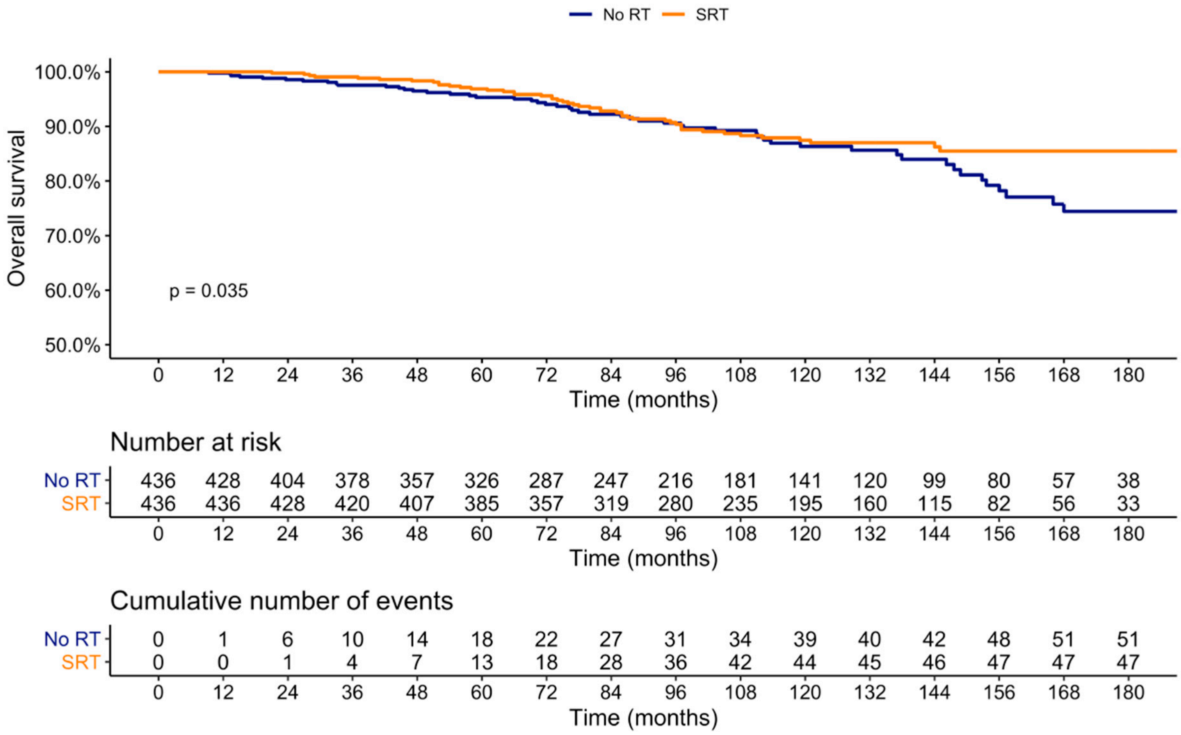

Figure S2. Kaplan-Meier plot depicting overall survival rates in prostate cancer patients treated with radical prostatectomy stratified according to observation vs. SRT for BCR, after 1:1propensity score matching, sensitivity analysis at six months.
